# Combined Exposure to Metals in Drinking Water Alters the Dopamine System in Mouse Striatum

**DOI:** 10.3390/ijerph18126558

**Published:** 2021-06-18

**Authors:** Haesoo Kim, Daeun Lee, Kisok Kim

**Affiliations:** Collage of Pharmacy, Keimyung University, Daegu 42601, Korea; 1004568@stu.kmu.ac.kr (H.K.); 1113180@stu.kmu.ac.kr (D.L.)

**Keywords:** arsenic, lead, cadmium, motor coordination, dopaminergic neurotransmission

## Abstract

Environmental exposure to arsenic (As), lead (Pb), and cadmium (Cd) frequently occurs; however, data on the specific effects of combined exposure on neurotransmission, specifically dopaminergic neurotransmission, are lacking. In this study, motor coordination and dopamine content, along with the expression of tyrosine hydroxylase (TH), dopamine transporter (DAT), vesicular monoamine transporter 2 (VMAT2), and dopamine receptors (DRs), were examined in the striatum of adult male mice following exposure to drinking water containing As, Pb, and/or Cd. We found that exposure to a metal mixture impaired motor coordination. After 4 weeks of treatment, a significant decrease in dopamine content and expression of TH, DAT, and VMAT2 was observed in the striatum of metal-mixture-treated mice, compared to the controls or single-metal-exposed groups. However, DRD1 and DRD2 expression did not significantly change with metal treatment. These results suggest that altered dopaminergic neurotransmission by the collective action of metals may contribute to metal-mixture-induced neurobehavioral disorders.

## 1. Introduction

Many people around the world are exposed to toxic metals in drinking water at concentrations that exceed the allowable limits determined by the World Health Organization [1]. Public health concerns surrounding water contamination include major toxic metal contaminants, such as arsenic (As), lead (Pb), and cadmium (Cd) [2]. Exposure to mixtures of these metals is a common and important determinant of toxicity [3]. The accumulation of these metals to toxic levels is associated with many diseases, including cancer, diabetes, respiratory disease, and cardiovascular diseases [4]. In addition, there is growing concern about the toxic effects of these metals on the central nervous system (CNS), as increasing evidence has revealed that the CNS is one of the major targets of As, Pb, and Cd [5,6]. Epidemiological studies have reported that chronic exposure to As through drinking water results in impaired development of intellectual function in children, as well as reduced sensory and motor function [7,8]. Likewise, Pb exposure is known to be related to cognitive impairment, executive function alterations, and fine motor control perturbations [9]. In addition, increased blood Cd is reported to be associated with worsened cognitive function and motor skills [10,11].

Although the mechanisms underlying the neurologic effects of these metal mixtures are unclear, studies on the neurotoxic consequences of exposure to a single metal have focused largely on neurotransmitter systems in the CNS. Studies in rodents have shown that single exposure to As, Pb, or Cd alters the dopaminergic system [1,12,13]. Rats treated with As had increased striatal dopamine content and upregulated mRNA levels of dopamine receptor (DR) D1 in the striatum [14]. Chronic exposure to Pb in drinking water was reported to induce neurobehavioral impairments and an altered dopaminergic system in rodents [15]. In addition, Cd treatment induced a significant reduction in dopamine (DA) in the cerebellum, hippocampus, and cerebral cortex of rats [16]. These observations suggest that the neurotoxic effects observed with combined exposure to As, Pb, and Cd result, at least partially, from an interference with dopaminergic neurotransmission.

As a neurotransmitter, dopamine plays major roles in many brain functions, including cognition, emotion, and control of locomotion [17]. Dopamine is synthesized from tyrosine by the action of tyrosine hydroxylase (TH), a rate-limiting enzyme in catecholamine synthesis. The action of dopamine is terminated by reuptake from the synaptic cleft into the presynaptic neuron through the dopamine transporter (DAT). Dopamine is then transported and packaged into vesicles for release by vesicular monoamine transporter 2 (VMAT2) [18]. Meanwhile, dopamine affects neurons through dopamine receptors (DRs), which can be divided into two receptor subtype families according to their biochemical and physiological properties: D1-like and D2-like. The D1-like receptor subtype includes D5 (DRD5), whereas the D2-like receptor subtype consists of D3 (DRD3) and D4 (DRD4) [19]. The dopamine receptor D1 (DRD1) interacts with G proteins to stimulate adenylate cyclase activity, whereas the dopamine receptor D2 (DRD2) inhibits adenylate cyclase activity [20]. 

To assess the effects of combined exposure to As, Pb, and Cd on dopaminergic neurotransmission in the mouse brain, we evaluated the motor coordination, dopamine content, and TH, DAT, VMAT2, and DRs expression in mice exposed to single or mixed metals. 

## 2. Materials and Methods

### 2.1. Animals

Male C57BL/6 mice (*n* = 40, 7 weeks old, weighing 22.0 ± 0.5 g) were randomly divided into five groups: water-treated control, As (5 mg/L), Pb (50 mg/L), Cd (0.5 mg/L), and metal-mixture-treated (5 mg/L As + 50 mg/L Pb + 0.5 mg/L Cd) groups. The treatment doses were determined based on previously conducted studies and WHO guideline values for drinking water [14,21,22]. After 1 week of acclimation, the metal-treated mice (*n* = 8 per group) received As, Pb, and Cd in the form of sodium arsenite, lead (II) acetate trihydrate, and cadmium chloride, respectively, in their drinking water for 4 weeks. Control animals were given only distilled deionized water. The animals were housed 3 per cage and maintained under standard conditions (room temperature: 20  ±  2 °C; relative humidity: 50  ±  5%) on a light:dark cycle of 12:12 h. Water consumption was measured throughout the study period and the mice were weighed weekly. After 4 weeks of administration, 3 mice in each group were used for dopamine measurement and the remaining 5 mice in each group were used for Western blot and real-time PCR analysis. Mice and metal were obtained from Samtako Laboratories (Osan, Korea) and Sigma-Aldrich (St. Louis, MO, USA), respectively. Other reagents were of the highest quality available and were purchased from commercial sources. Experiments were approved by the Institutional Animal Care and Use Committee of Keimyung University, Korea (approval No.: KM 2020-002). Experiments were conducted according to NIH guidelines for the care and use of laboratory animals. Animals were housed in a specific pathogen-free (SPF) facility with free access to food and water. At the end of the treatment regimen, mice were sacrificed by CO_2_ asphyxiation, and the brains were immediately collected. Striatum were dissected from the brains and stored at −70 °C until use.

### 2.2. Motor Coordination

Motor coordination of mice was assessed using a rota-rod treadmill (Jeungdo Bio & Plant, Seoul, Korea). The mice were trained on the rota-rod treadmill for 3 days before the experiment. The accelerating speed of the rota-rod was set to increase from 10 rpm to 30 rpm over 10 s. The animals were placed on the treadmill and the time at which each mouse fell off the rod was recorded. The average latency time of three trials was used for analysis.

### 2.3. Measurement of Dopamine

Dopamine content was analyzed by rapid dissection of the striatum sample from each brain on ice. The striatal tissue was used to analyze dopamine concentration of either control or metal-exposed mice using the mouse dopamine ELISA kit (Cusabio, Carlsbad, CA, USA) following the manufacturer’s instructions. The absorbance of each well was measured at a wavelength of 450 nm using a microplate reader (Infinite 200 pro, Tecan, Männedorf, Switzerland). The detection range and sensitivity of this assay are 5–1000 pg/mL and 2.5 pg/mL, respectively.

### 2.4. Real-Time PCR Analysis

For the quantification of mRNA levels, total RNA was extracted from the striatum using NucleoSpin RNA kit (Macherey-Nagel, Germany) according to the manufacturer’s protocol. RNA samples were quantified using a NanoDrop 2000 spectrophotometer (Thermo Scientific, Waltham, MA, USA) and total RNA from each sample was reverse-transcribed using an iScript cDNA Synthesis kit (Bio-Rad, Hercules, CA, USA) according to the manufacturer’s protocol. The cDNAs were characterized using SsoAdvanced Universal SYBR Green Supermix kit (Bio-Rad) on a CFX96 real-time PCR system (Bio-Rad). The following genes were analyzed: TH (NM_009377.1), DAT (NM_010020.3), VMAT2 (NM_172523.3), DRD1 (NM_010076), DRD2 (NM_010077), and the reference gene β-actin (NM_007393) (Table 1). β-actin, a housekeeping gene, was used for normalization. Data were analyzed using Cq number. The Cq data gained from the PCR amplification were analyzed with CFX Manager Software (Bio-Rad) to obtain the expression level of amplified genes.

### 2.5. Western Blot Analysis

For Western blotting, the striatum was homogenized in radioimmunoprecipitation assay (RIPA) buffer (Sigma-Aldrich, St. Louis, MO, USA) containing 1% protease inhibitor cocktail and a phosphatase inhibitor cocktail. The homogenate was centrifuged (20 min, 4 °C), and the supernatant was collected. The protein concentration was estimated using the Bradford method, with bovine serum albumin (BSA) as the standard. An aliquot of 5 μg protein was subjected to 10% sodium dodecyl sulfate-polyacrylamide gel electrophoresis (SDS-PAGE), and the fractionated proteins were then transferred onto a nitrocellulose membrane. For immunoblotting, the following primary antibodies were used: mouse anti-TH monoclonal antibody (1:150,000 dilution; Chemicon, Temecula, CA, USA), rat anti-DAT monoclonal antibody (1:500 dilution; Santa Cruz Biotechnology, Santa Cruz, CA, USA), mouse anti-VMAT2 monoclonal antibody (1:1000 dilution; Santa Cruz Biotechnology), mouse anti-DRD1 monoclonal antibody (1:500 dilution; Santa Cruz Biotechnology), and mouse anti-DRD2 monoclonal antibody (1:1000 dilution; Santa Cruz Biotechnology). After incubation with horseradish peroxidase (HRP)-conjugated secondary antibodies (Santa Cruz Biotechnology), the immunoreactive bands were visualized using enhanced chemiluminescence (ECL) Western blotting detection reagents (Amersham Biosciences, Piscataway, NJ, USA) and X-ray film. Band intensity was measured using the ImageJ program (NIH, Bethesda, MD, USA). β-actin was used as a control for immunoblotting.

### 2.6. Statistical Analysis

Motor coordination performance, dopamine concentration, mRNA expression level, and densitometric measurements of protein expression in metal-treated mice were compared with those of the controls using one-way analysis of variance (ANOVA), followed by a post hoc Duncan test. The assumptions of normality and homogeneity of variances were tested using Kolmogorov–Smirnov test and Levene’s test for homogeneity, respectively. *p* values < 0.05 or <0.01 were deemed statistically significant. All statistical analyses were conducted using SAS v. 9.4 statistical software (SAS Institute Inc., Cary, NC, USA).

## 3. Results

### 3.1. Body Weight

**Figure 1:**
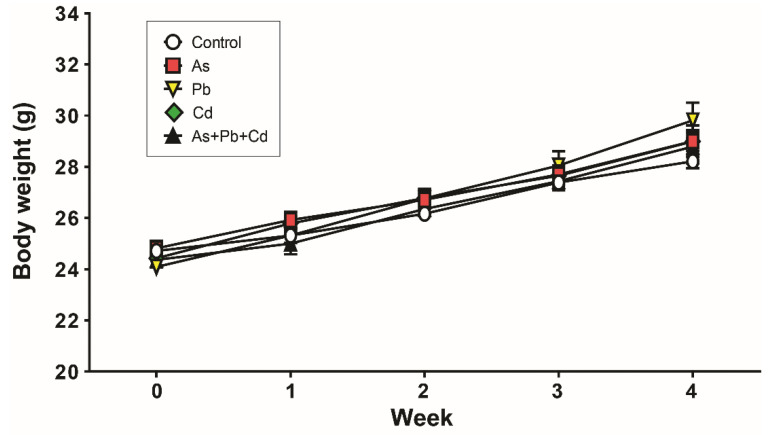
Effects of metal treatment on body weight. Values are expressed as the mean ± standard deviation (SD) for each group (*n* = 8).

Water consumption was not significantly different among groups. The mean As, Pb, and Cd intakes from drinking water were equivalent to approximately 0.77, 10.26, and 0.07 mg/kg body weight per day, respectively. The body weight of metal-treated animals did not significantly differ from that of the controls during 4 weeks of treatment (Figure 1).

### 3.2. Motor Coordination

**Figure 2:**
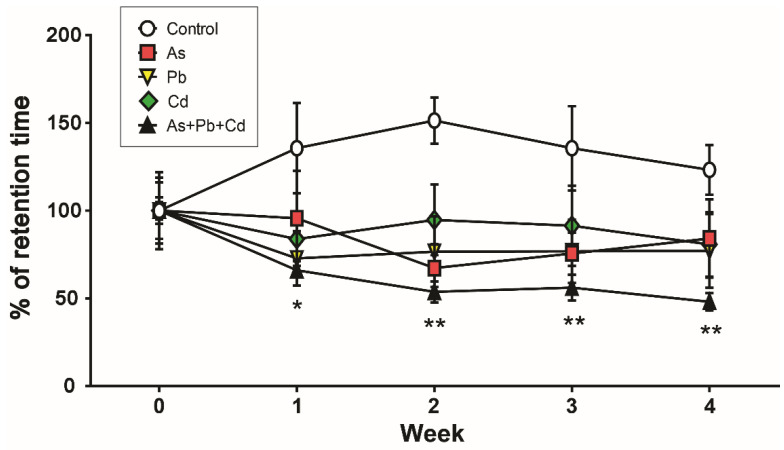
Effects of metal on rota-rod task performance. The time of mice remaining on the rotating rod was expressed as a % of retention time starting before treatment (week 0). Error bars reflect standard error of the mean (SEM) for each group (*n* = 8). * *p* < 0.05, ** *p* < 0.01, compared with the control group.

Motor coordination is expressed as a percentage of retention time (Figure 2). The absolute retention times were not significantly different across treatment groups before treatment (week 0). The retention time of the control mice increased slightly after week 0 of treatment. Metal-treated mice displayed reduced retention time on the accelerating rotating rod over the experimental timeframe. Beginning with the first week of treatment, a significant decrease in retention time was observed in metal-mixture-treated mice compared to control mice (*p* < 0.05 or *p* < 0.01). There was no significant difference in retention time on the rota-rod among single metal-treated groups throughout the study.

### 3.3. Dopamine Content

**Figure 3:**
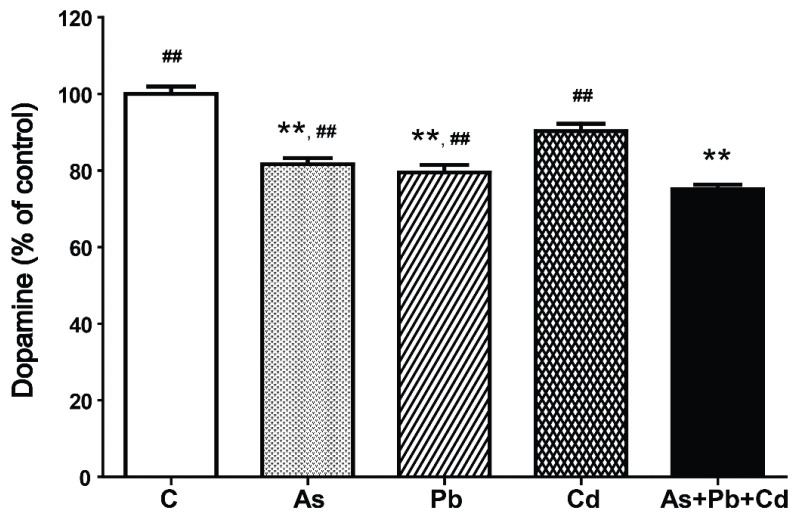
Relative dopamine content in the striatum of mice treated with As, Pb, and Cd. Values represent the means ± standard deviation (SD) of each group (*n* = 3). ** *p* < 0.01, compared with the control group. ^##^ *p* < 0.01, compared with the metal mixture (As + Pb + Cd) group.

To understand the effect of metal on dopamine synthesis, we examined striatal dopamine levels following exposure to single or mixed metals (Figure 3). After 4 weeks of treatment, the dopamine level decreased significantly in mice treated with As, Pb, or a metal mixture compared with controls (*p* < 0.01). The dopamine content was significantly lower in mice treated with a metal mixture compared with single-metal-treated mice (*p* < 0.01).

### 3.4. Gene Expression in the Striatum

**Figure 4:**
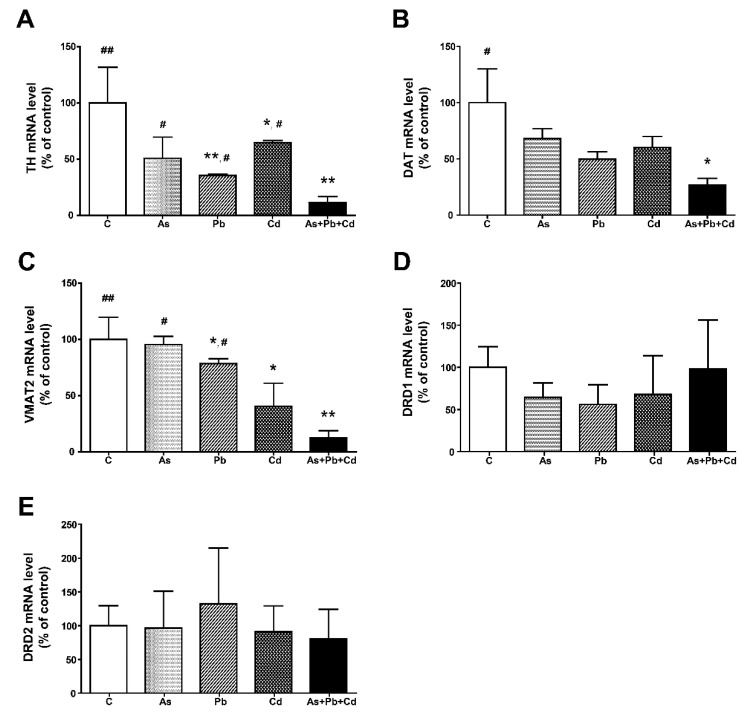
Relative gene expression levels of tyrosine hydroxylase (TH) (**A**), dopamine transporter (DAT) (**B**), vesicular monoamine transporter 2 (VMAT2) (**C**), dopamine receptor D1 (DRD1) (**D**), and dopamine receptor D2 (DRD2) (**E**) in the striatum of mice. Error bars reflect standard error of the mean (SEM) of each group (*n* = 5). * *p* < 0.05, ** *p* < 0.01, compared with the control group. ^#^ *p* < 0.05, ^##^ *p* < 0.01, compared with the metal mixture (As + Pb + Cd) group.

The molecular basis of motor coordination deficits was elucidated by comparing differences in mRNA expression of TH, DAT, VMAT2, DRD1, and DRD2 between controls and metal-treated mice using real-time RT-PCR. Striatal TH gene expression decreased significantly in mice treated with Pb, Cd, or a metal mixture compared with controls (*p* < 0.05 or *p* < 0.01; Figure 4A). TH gene expression was also significantly lower in the striatum of mice treated with a metal mixture compared to the striatum of mice treated with As, Pb, or Cd (*p* < 0.05). DAT gene expression was significantly reduced in metal-mixture-treated mice compared with control mice; however, the expression of DAT did not significantly change with single-metal treatment (Figure 4B). In the striatum, VMAT2 mRNA expression decreased significantly in mice treated with Pb, Cd, or a metal mixture compared with controls (*p* < 0.05 or *p* < 0.01; Figure 4C). VMAT2 gene expression decreased significantly in mice treated with metal mixture compared with mice treated with As or Pb (*p* < 0.05). Treatment with single or mixed metals had no significant overall effects on DRD1 and DRD2 mRNA expression in the striatum of treated mice (Figure 4D,E).

### 3.5. Protein Expression in the Striatum

**Figure 5:**
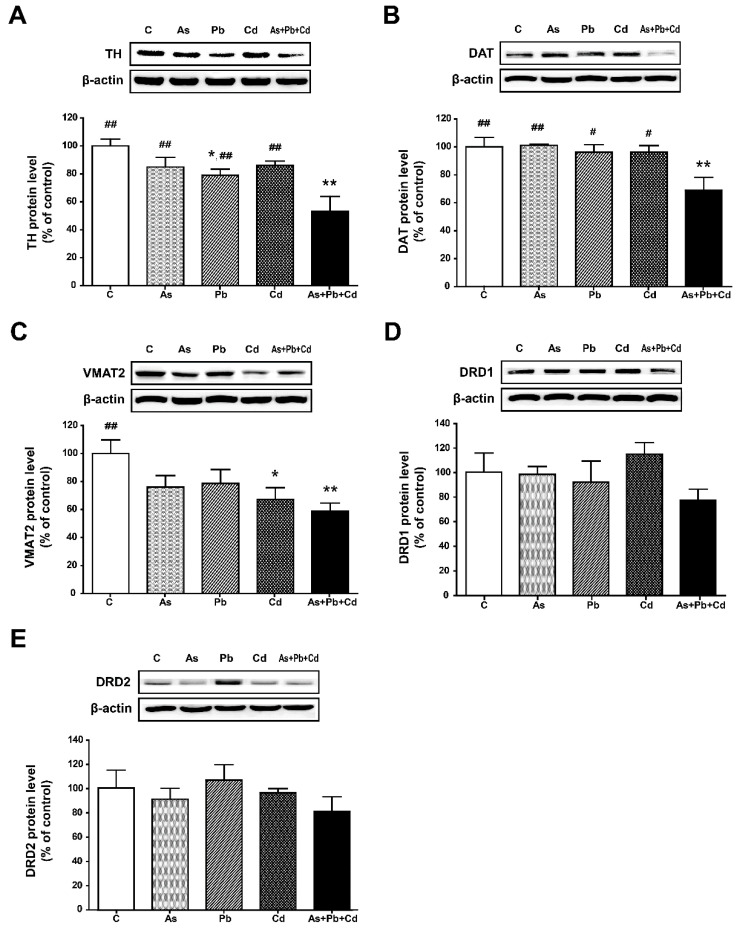
Relative protein expression levels of tyrosine hydroxylase (TH) (**A**), dopamine transporter (DAT) (**B**), vesicular monoamine transporter 2 (VMAT2) (**C**), dopamine receptor D1 (DRD1) (**D**), and dopamine receptor D2 (DRD2) (**E**) in the striatum of mice. Each panel shows a representative Western blot (**top**) and densitometric analysis of bands in the control and metal-treated groups (**bottom**). Values represent the means ± standard deviation (SD) of each group (*n* = 5). * *p* < 0.05, ** *p* < 0.01, compared with the control group. ^#^ *p* < 0.05, ^##^ *p* < 0.01, compared with the metal mixture (As + Pb + Cd) group.

Based on the mRNA data, we examined the protein levels of TH, DAT, VMAT2, DRD1, and DRD2 using Western blotting. Representative Western blots of protein expressions and quantitative analysis of optical densities are shown in Figure 5. As with the mRNA analysis, exposure to the metal mixture significantly decreased protein the expression of TH, DAT, and VMAT2 (*p* < 0.01; Figure 5A–C). Exposure to mixed metals also significantly lowered the protein expression of TH and DAT, compared to exposure to a single metal (*p* < 0.05 or *p* < 0.01). Consistent with the mRNA results, protein levels of DRD1 and DRD2 did not significantly change with the treatment of single or mixed metals (Figure 5D,E).

## 4. Discussion

Pb, Cd, and As are major toxic metals in the environment that accumulate simultaneously in the general population and cause various neurobehavioral deficits [23]. Chronic Pb exposure has been reported to impair motor coordination using a rota-rod test in male rats and gestational exposure to Cd reduced locomotor activity in rats [21,24]. An association between As exposure in drinking water and impaired motor function in children was demonstrated in an epidemiological study [25]. 

There are many studies reporting behavioral effects following individual metal exposure; however, data are lacking for multi-metal exposure. Here, we find individual exposure to As, Pb, or Cd decreased motor coordination slightly compared to the control group, while combined exposure to these three metals significantly reduced motor coordination compared to the control mice. A previous study demonstrated that a mixture of heavy metals induced synergistic neurotoxicity, leading to impairments in neurobehavioral functions [26]. The results of our study also suggest that the combined exposure to metals can collectively cause the impairment of motor function.

The present study indicates that there are changes to motor coordination, resulting in dopaminergic neurotransmission disturbances. Previous studies have also reported behavioral effects of metals caused by changes in the neurotransmitter system [27,28,29]. Dopamine is known to play a critical role in the regulation of motor activity [30,31]. In this study, dopamine levels in mouse brain decreased in groups exposed to a single metal compared to the control group. The dopamine level in the mixed-metal-exposed group significantly decreased compared to the control and the single-metal-exposed groups. A previous study on the neurotoxin effects of metal mixture found dopamine levels significantly reduced in the brains of rats simultaneously exposed to As, Pb, and Hg and moderately reduced in the brains of rats individually exposed to As, Pb, and Hg [32]. Together, these findings suggest that combined exposure to metals collectively reduces dopamine content in the stratum compared to single-metal exposure. The results of our dopamine content analysis are consistent with the motor coordination findings, suggesting that a change in motor coordination following metal exposure is closely related to dopamine levels in the striatum of mice. 

Dopamine is synthesized from tyrosine by TH, a rate-limiting enzyme in the biosynthesis of dopamine [33]. Therefore, we examined whether metal exposure modulates the TH gene and protein expression in the striatum. Several studies have reported an association between TH expression and exposure to As, Pb, or Cd [34,35,36,37]; however, few reports have studied the effect of combined metal exposure on TH expression. In this study, similar patterns of gene and protein expression of TH were found in the striatum. The expression of TH was significantly reduced in mice exposed to a metal mixture compared to mice exposed to individual metals. This finding is consistent with the results of dopamine analysis, suggesting that reductions in dopamine concentrations may be caused by the downregulated expression of TH. The expression of TH may play an important mechanistic role in the neurobehavioral toxicity induced by exposure to combined metals.

Several major markers in the striatum were also studied to more fully understand the mechanism underlying metal-mixture-induced neurotoxic effects on dopaminergic neurotransmission. We found that combined exposure to As, Pb, and Cd downregulated the expression of DAT and VMAT2 in the striatum. The expression of DRD1 and DRD2 was not significantly different between single metal or combined metal exposure. 

Several studies have reported on the relationship between metal exposure and DAT or VMAT2 [38,39,40]. DAT is an integral membrane protein whose main function is reuptake of released DA from the synaptic cleft [41,42,43], while VMAT2 is responsible for transporting monoamine neurotransmitters, including dopamine, into synaptic vesicles [44]. Therefore, the downregulation of DAT or VMAT2 would affect dopaminergic neurotransmission and may be associated with several neurobehavioral disorders. 

Mixed exposure to metals may affect the processes of absorption, distribution, metabolism, and excretion of each metal, leading to metal–metal interactions, such as competition, antagonism, and synergism [45]. In this study, combined exposure to As, Pb, and Cd significantly reduced dopamine content and TH expression compared with exposure to individual metals. We found significant decreases in DAT and VMAT2 expression in the striatum, while the expression of DRs did not change significantly in the striatum of metal mixture-exposed mice. DA is known as a main neuromodulator for motor activity, and the major functional target of dopamine is the striatum in the midbrain [46]. Therefore, the abnormality of motor coordination caused by the combined exposure to metals would be closely related to the decrease in TH expression and dopamine amount. In addition, the results of this study suggest that DAT and VMAT2 may also play an important role in neurobehavioral toxicity, including the deterioration of motor coordination.

## 5. Conclusions

Our findings indicate that the altered homeostasis of striatal dopamine is closely related to TH, DAT, and VMAT2 expression, and may contribute to metal-mixture-induced neurobehavioral toxicity, including impaired motor coordination. Further studies are needed to fully elucidate the effects of a metal mixture on dopaminergic signaling, including its effects on transporter and receptor activity. Clarification is also needed to determine whether neurobehavioral changes caused by exposure to a metal mixture are due to synergistic, additive, or antagonistic effects of the individual metals.

## Figures and Tables

**Table 1:** Primer sequences (5′ to 3′) and NCBI references.

Gene	NCBI Ref Seq Number	Sense	Antisense
TH	NM_009377.1	CAGCTGGAGGATGTGTCTCA	GGCATGACGGATGTACTGTG
DAT	NM_010020.3	TTGCAGCTGGCACATCTATC	ATGCTGACCACGACCACATA
VMAT2	NM_172523.3	ATGTGTTCCCGAAAGTGGCA	AAGTTGGGAGCGATGAGTCC
DRD1	NM_010076	GGAAGATGCCGAGGATGA	TAGATACTGGTGTAAGTGACGAT
DRD2	NM_010077	CTCAACAACACAGACCAGAAT	GAACGAGACGATGGAGGA

## Data Availability

The data used to support the findings of this study are available from the corresponding author upon request.
